# Dietary quality and adherence to dietary recommendations in Chinese patients with chronic kidney disease

**DOI:** 10.3389/fnut.2025.1547181

**Published:** 2025-02-03

**Authors:** Wenwei Ouyang, Bingjie Xiao, Huifen Chen, Lizhe Fu, Fang Tang, Gaetano Marrone, Xusheng Liu, Yifan Wu, Juan Jesús Carrero

**Affiliations:** ^1^Department of Global Public Health, Karolinska Institute, Stockholm, Sweden; ^2^Key Unit of Methodology in Clinical Research, The Second Affiliated Hospital of Guangzhou University of Chinese Medicine (Guangdong Provincial Hospital of Chinese Medicine), Guangzhou, China; ^3^The Second Clinical College of Guangzhou University of Chinese Medicine, Guangzhou, China; ^4^Chronic Disease Management Outpatient, The Second Affiliated Hospital of Guangzhou University of Chinese Medicine (Guangdong Provincial Hospital of Chinese Medicine), Guangzhou, China; ^5^Department of Nephrology, The Second Affiliated Hospital of Guangzhou University of Chinese Medicine (Guangdong Provincial Hospital of Chinese Medicine), Guangzhou, China; ^6^Department of Medical Epidemiology and Biostatistics, Karolinska Institute, Stockholm, Sweden

**Keywords:** chronic kidney disease, nutrient, dietary quality, guideline, adherence

## Abstract

**Objectives:**

There is a lack of data regarding the quality of the diet and the adherence to dietary guidelines of patients with non-dialysis-dependent CKD (NDD-CKD) in China.

**Design and methods:**

Single-center cross-sectional study of 261 patients with CKD stages 3–5, who responded to 3-day dietary records and undertook 24-h urine samples along with clinical, laboratory, and anthropometric assessments. We compared their food intake with Chinese recommendations for CKD patients, assessed dietary quality through the Chinese Healthy Eating Index (CHEI), and calculated the contribution to energy intake by processed foods according to the NOVA classification.

**Results:**

Average energy intake was 30 ± 9 Kcal/kg/d, and 65% consumed less energy than recommended. The average protein intake was 1.2 ± 0.5 g/Kg/d, and 81% consumed more than recommended. 71% of patients consumed excess sodium and 80% consumed too little fiber. These proportions worsened across more severe CKD stages (all P trend value <0.05). The diet was considered of moderate quality (CHEI score 59.5 ± 11.0), and patients with CKD stages 4–5 scored progressively worse (P trend = 0.008). Total grains and tubers supplied 50 and 30% of the total energy and protein intake, respectively. Processed and ultra-processed foods contributed to 23.3% of dietary energy and 11.7% of food weight.

**Conclusion:**

A large proportion of NDD-CKD at our center showed low adherence to diet recommendations. Although consumption of processed foods was low, diet quality worsened with more severe CKD, with low intake of whole grains, dairy, and soybean.

## 1 Introduction

Chronic kidney disease (CKD) is a major global health problem, and 10.8% of adults in China are estimated to suffer from it (1, 2). CKD is associated with poor outcomes and high medical costs, especially when patients progress to end-stage kidney disease (ESKD) (3). Given the kidney’s unique role in maintaining nutrient homeostasis, people with CKD need dietary modifications to avoid kidney overload, delay progression, and manage CKD complications related to water, electrolyte, and acid–base imbalance (4).

Various clinical guidelines provide dietary recommendations for patients with CKD (5–9). Specifically in China, the National Health Commission of the People’s Republic of China in 2017 (5), and the Chinese Nephrology Association in 2021 emphasize target intakes for these patients’ energy, macro and micronutrients (6). Further, there is a need to move single nutrient recommendations to dietary patterns and diet quality (9), as nutrients are not only eaten in isolation, but as part of foods and associated with cultural traditions, availability, and affordability (10, 11). Observational studies consistently show that a healthy dietary pattern is associated with lower ESKD risk and lower mortality risk in patients with moderate to advanced CKD (12, 13). Unhealthy diets are typically characterized by high energy density, added sugar, sodium, and fat, along with low fiber and high consumption of ultra-processed food (UPF) patterns (14, 15). Epidemiological evidence has suggested that higher UPF consumption is associated with a higher risk of incident CKD (16, 17), and a more rapid eGFR decline in the general population (18).

Various studies, predominantly from Europe and America (19–21), but also from Asia and African countries like Japan, Qatar, and Ethiopia (22–24), all suggest that a considerable proportion of patients do not follow CKD-specific dietary guidelines. A recent survey from the International Society of Nephrology revealed significant gaps in global kidney nutrition care service capacity, availability, cost coverage, and deficiencies in interdisciplinary communication on kidney nutrition care delivery, especially in lower-income countries (25). In China, the food structure and eating habits are different from many countries in the world, and it is unknown how the diet quality of patients with CKD is, and to what extent CKD-specific dietary recommendations are followed. We explored these aspects in routinely caring for patients with non-dialysis-dependent CKD (NDD-CKD) from a single center in China.

## 2 Materials and methods

### 2.1 Study design and population

This was a cross-sectional study nested within the Self-Management Program for Patients with Chronic Kidney Disease (SMP-CKD) Study. The SMP-CKD Study is an ongoing ambispective cohort study to access self-management components and disease progression in patients with CKD managed at the nephrology department at the Chronic Disease Management Center at the Guangdong Provincial Hospital of Chinese Medicine (GPHCM) in Guangdong province, China. The design and methods for selecting study participants have been previously described (26). The study protocol was approved by the Ethics Committee of the Guangdong Provincial Hospital of Chinese Medicine (No. 2019–153-01). A written informed consent from patients is required before commencing.

Patients were eligible for inclusion in this study if they were Chinese citizens, aged 18 to 80 years, diagnosed with CKD stage 3–5, and received at least 3 months of nutritional counseling. CKD is defined by a glomerular filtration rate (GFR) < 60 mL/min/1.73 m2 or markers of kidney damage, or both, of at least 3 months duration (27). GFR was calculated using the CKD-EPI equation, and Kidney Disease Outcomes Quality Initiative (KDOQI) guidelines were used to define the CKD stage (28). Patients were excluded if (1) they had not completed a dietary assessment; (2) had extreme values of self-reported energy intake (women: <500 or > 3,500 kcal/d; men<700 or > 4,500 kcal/d); (3) withdrew their informed consent to participate. After applying these inclusion and exclusion criteria, 261 patients were included in our analysis. The patient selection flow can be found in Supplementary Figure 1.

### 2.2 Data collection

Patients were asked to fill out a predetermined questionnaire to collect their social-demographic data (e.g., age, sex, marital status, education level, and insurance type), lifestyle habits (e.g., smoking, alcohol), and history of selected diseases (e.g., hypertension, diabetes, and cardiovascular diseases). Diagnostic and pathological data were obtained from the electronic healthcare records (EHRs) at GPHCM. Information on medication use was combined with data from the questionnaire and EHRs.

Biochemical assays were performed at the hospital’s central laboratory using standardized methods. Venous blood samples were used to quantify levels of creatinine (Scr), blood urea nitrogen (BUN), Serum albumin (ALB), and total cholesterol (TC). A 24-h urine samples were also collected. The 24-h urine sodium excretion and urine urea nitrogen (UUN) were evaluated from 24-h urine samples. The sodium concentrations in mmol/l were multiplied by the urine volume in 24 h to obtain values in mmol per 24 h. The estimated protein intake (EPI) was calculated from 24-h UUN using the following equation: 6.25 × (UUN+(weight×0.031)) (29). Anthropometric measurements such as body height, weight, fat mass, and fat percent were estimated by a bioimpedance analysis (InBody770, InBody, Guangzhou, China). Body mass index (BMI) was then calculated as weight (kg) divided by height squared (m2). Ideal body weight (IBW) was calculated by the Broca-Katsura formula (30, 31), which is IBW = (height in cm-100) *0.9 for men and IBW = (height in cm-100) *0.9–2.5 for women. Protein-energy wasting (PEW) was diagnosed based on four distinct categories: (1) biochemical indicators, (2) low body weight, reduced body fat or weight loss, (3) decreased muscle mass, and (4) low protein or energy intake proposed by ISRNM (32).

### 2.3 Dietary records

Patients’ dietary intake was assessed through 3-day dietary records. All foods and beverages consumed on three consecutive days, two on weekdays and one weekend day were recorded. Dieticians in the Chronic Disease Management Center in GPHCM trained the patients on how to report their diet in the diaries. Records included the type of food, the amount and when it was eaten, etc. All types and amounts of food and liquid ingested were reported in predefined portion sizes (gram, Liang or cup, 1 Liang = 50 g, 1 cup = 200 mL). To ensure the accuracy of the records, a weight device and food picture models were also provided to help them estimate portion sizes. Food records were reviewed in detail by trained staff in the center, and if there were obvious errors, patients were asked to check their records again. Dietary records were then converted into daily nutrient intakes by using the 2009 and 2018 Chinese Food Composition Tables (33, 34).

### 2.4 Adherence to nephrology dietary guideline recommendations

The patient’s adherence to guideline recommendations was assessed by comparing the consumed foods reported in the 3-day dietary records with the 2017 Dietary Guide for Chronic Kidney Disease Patients (WS/T 557–2017) (5). This standard specifies the principles of dietary guidance, recommended intake of energy and other nutrients, development of dietary prescriptions, and monitoring and assessment of nutritional intake for patients with chronic kidney disease. Table 1 summarizes these recommendations.

**Table 1:** Daily intake recommendations to Chinese patients with CKD stages 3–5.

Nutrients	Recommended daily intake
DEI [KJ (Kcal)/Kg of IBW]	Age below 60: >146KJ (35Kcal);Age above 60: 126-146KJ (30–35Kcal)
DPI [g/Kg of IBW]	0.6–0.8 g/Kg
Carbohydrate [% of energy]	55–65%
Fat [% of energy]	25–35%
Saturated fat [% of energy]	<10%
Trans-fatty acids [% of energy]	<1%
Fibre [g]	>14 g/4180 kJ (1,000 kcal)
Sodium [mg]	<2000 mg/d
Phosphorus [mg]	<800 mg/d

### 2.5 Dietary quality and consumption of processed/ultra-processed foods

The Chinese Healthy Eating Index (CHEI) was applied to assess the quality of the diet in terms of dietary components. A detailed description and validation of CHEI are provided elsewhere (35, 36). Briefly, the CHEI consists of 17 components, 12 of which assess whether the dietary intake is adequate, including total grains, whole grains and mixed beans, tubers, total vegetables, dark vegetables, fruits, dairy, soybeans, fish and seafood, poultry, eggs, seeds, and nuts. The other five components assess the limitations of diet: red meat, cooking oils, sodium, added sugars, and alcohol. The daily food and nutrient intakes were transformed into standard potions (SP) based on the 2016 Dietary Guidelines for Chinese (DGC-2016) (6). For these items, zero intake and recommended SP intake or above would, respectively, get a zero point or full point (5 or 10 points), while intermediate intakes between zero and the relevant recommended value would award a score according to the formula (score = (actual intake/recommended intake) × full point). The scoring of limitation components is the opposite of the scoring of adequacy components. The total scores of CHEI range from 0 to 100. A higher score means a better adherence to DGC-2016. Since no data on added sugars existed in the Chinese Food Composition Database, the component was calculated using the USDA database (37). More details can be found on Supplementary Table 1.

The consumption of processed foods was estimated by the NOVA classification system. The NOVA classification system includes four groups: unprocessed or minimally processed foods, processed culinary ingredients, processed foods (PF), and ultra-processed foods (UPF) (14, 38). More than 900 food items reported in 3-day dietary records were classified according to NOVA, and uncertain food items were discussed among coauthors by reviewing their food ingredients to reach a consensus. This study primarily focused on processed and ultra-processed food groups. The energy contribution and weight proportion of these two types of foods in the diets were then calculated.

### 2.6 Statistical analysis

The variables were assessed for normality of distribution. Continuous variables were expressed as means ± standard deviation (SD) or median (inter quartile range) as appropriate. Categorical variables were presented as percentages. The analyses were conducted in all patients and stratified by CKD stage and sex. The trend of the nutrients and dietary quality score among CKD stages was evaluated using the Cuzick trend test, and we did not account for the probability of chance findings by multiple comparisons. The comparison between sex was done by Student-t test or Mann–Whitney U test. All the analyses were performed in STATA MP 14.0 (Stata Corporation, College Station).

## 3 Results

### 3.1 Patient characteristics

Table 2 summarizes the clinical, demographic, and biochemical characteristics of the patients included in our study. A total of 261 patients with NDD-CKD stages 3–5 were enrolled, with a mean age of 58 ± 13 years and 52% were male. The average eGFR was 31 ± 17 mL/min/1.73 m2. Diabetes and cardiovascular disease were present in 19% and 11% of patients, respectively. The primary glomerulonephritis accounted for 42% of the CKD. PEW was presented in 13% of the patients.

**Table 2:** Clinical, demographic, and biochemical characteristics of study participants (*n* = 261).

Variable	Total	CKD-3 (*n* = 130)	CKD-4 (*n* = 67)	CKD-5 (*n* = 64)
Age, years	58 ± 13	59 ± 12	57 ± 13	57 ± 12
Male, n (%)	136 (52)	69 (53)	40 (60)	27 (42)
Han Nationality, n (%)	259 (99)	127 (99)	63 (97)	60 (100.0)
Education level, n (%)				
Primary school or below	36 (14)	14 (11)	10 (15)	12 (19)
Middle or high school	155 (60)	74 (57)	42 (63)	39 (61)
University or above	70 (27)	42 (32)	15 (22)	13 (20)
Have medical insurance, n (%)	161 (62)	81 (62)	43 (64)	37 (58)
Current Smoker, n (%)	58 (22)	26 (20)	20 (30)	12 (19)
Body Mass Index, BMI, Kg/m^2^	22.7 ± 3.5	23.5 ± 3.5	22.2 ± 2.9	21.6 ± 3.7
Estimated GFR, mL/min/1.73 m^2^	31 ± 17	46 ± 9	23 ± 4	10 ± 4
BUN, mg/dL	32 ± 15	24 ± 7	35 ± 12	47 ± 17
Serum Albumin, g/dL	4.0 ± 1.3	4.0 ± 1.3	4.1 ± 1.1	3.8 ± 1.5
Pathological type, n (%)				
PGN	109 (42)	53 (41)	30 (45)	26 (41)
SGN	118 (45)	58 (45)	28 (42)	32 (50)
TIN	8 (3)	5 (4)	1 (2)	2 (3)
HER	7 (3)	4 (3)	1 (1.5)	2 (3)
Unknown	19 (7)	10 (8)	7 (10)	2 (3)
Blood pressure, mm Hg				
Systolic	129 ± 15.4	126 ± 15	130 ± 14	133 ± 15
Diastolic	74 ± 10	73 ± 10	76 ± 9	74 ± 10
Diabetes mellitus, n (%)	50 (19)	23 (18)	14 (21)	13 (20)
Cardiovascular disease, n (%)	26 (11)	15 (13)	5 (8)	6 (11)
Medications, n (%)				
ACEi/ARBs	113 (43)	74 (57)	23 (34)	16 (25)
Beta blockers	69 (26)	28 (22)	20 (30)	21 (33)
Statins	82 (31)	46 (35)	21 (31)	15 (23)
Diuretics	21 (8)	5 (4)	5 (8)	11 (17)
PEW, n (%)	35 (13)	14 (11)	10 (15)	11 (17)

PGN, primary glomerulonephritis; SGN, secondary glomerulonephritis; TIN, tubulointerstitial nephritis; HER, hereditary glomerular diseases; BUN, blood urea nitrogen; ACEi, angiotensin-converting-enzyme inhibitors; ARBs, angiotensin receptor blockers; PEW, protein-energy wasting.

### 3.2 Daily intake of nutrients and adherence to guidelines

**Figure 1:**
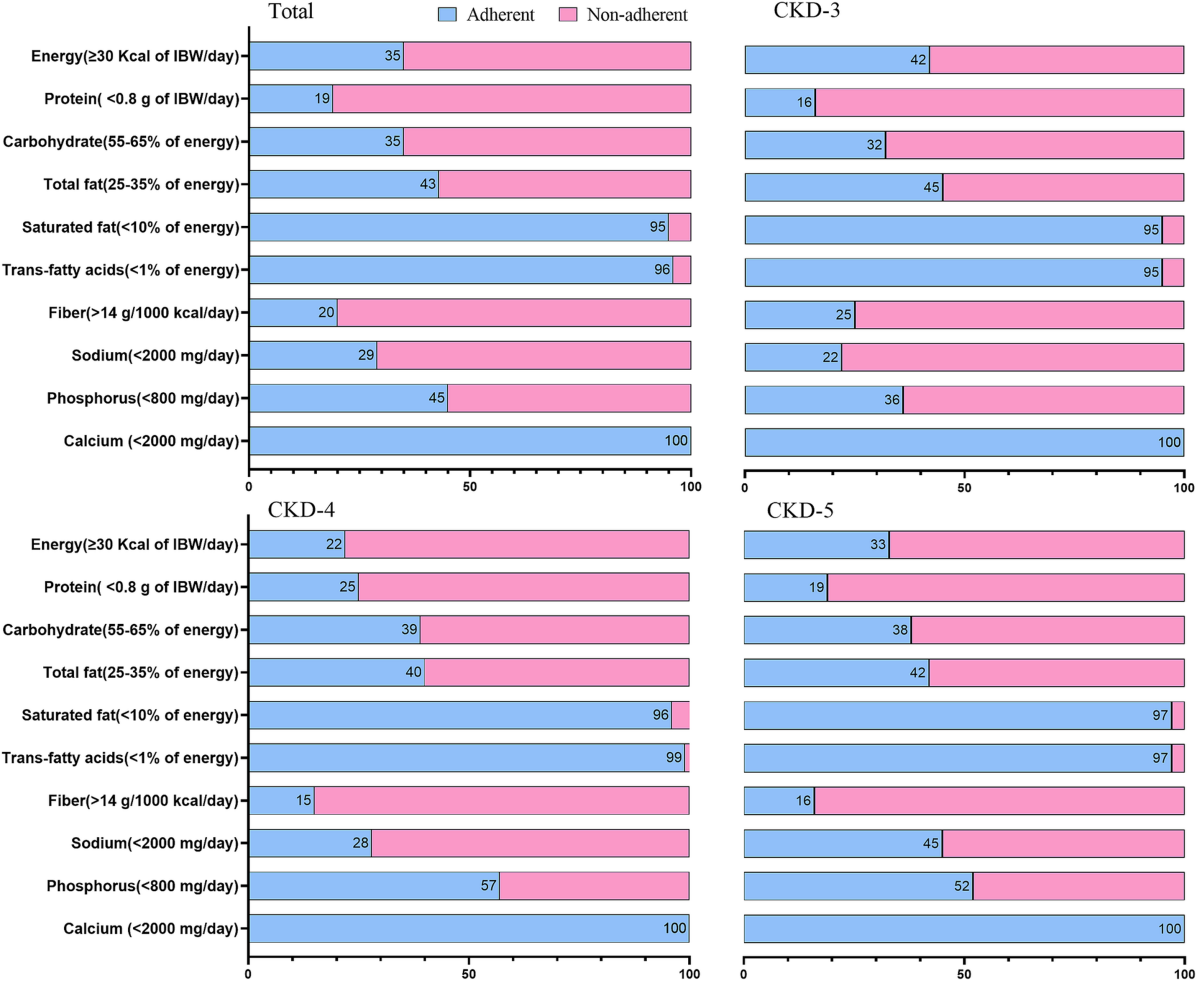
Proportion of patients with CKD stages 3–5 adhering to the Chinese dietary guide for chronic kidney disease. Data are expressed as percentage.

Table 3 and Supplementary Table 2 show the daily intakes of macro-and micronutrients. Figure 1 and Supplementary Figure 2 present the CKD stage-and sex-stratified proportions of individuals achieving the recommended intake as per national guidelines following 3 months of nutrition counseling. Supplementary Table 3 presents adherence to DEI and DPI recommendations by the presence or absence of PEW. The average daily energy intake (DEI) was 30 ± 9 Kcal/kg, and 35% of patients achieved the minimum DEI recommended. Only 14% of PEW patients achieved the minimum DEI recommended. The average daily protein intake (DPI) was 1.2 ± 0.5 g/Kg/d. More than 81% of patients consumed more protein than recommended. The estimated protein intake from 24-h UUN was similar (1.1 ± 0.4 g/Kg/d). The daily fiber intake was 10 ± 5 g and only 20% of individuals consumed sufficient fiber as dictated by guidelines. We observed lower DEI, DPI, fiber intake across more severe CKD stages (all *P* trend<0.05), and lower DEI, DPI in male patients (all *p* < 0.05).

**Table 3:** Daily intakes of macronutrients and micronutrients, overall and by stages of CKD severity.

Nutrients	Total (*n* = 261)	CKD-3 (*n* = 130)	CKD-4 (*n* = 67)	CKD-5 (*n* = 64)	*P* trend
Macronutrients					
Energy, *kcal*	1,602 ± 491	1,660 ± 509	1,582 ± 430	1,508 ± 515	<0.01
DEI, kcal/kg/d	30 ± 9	31 ± 10	29 ± 7	28 ± 9	0.03
Total protein*, g*	62 ± 21	67 ± 23	59 ± 19	56 ± 17	<0.01
DPI*, g/Kg/d*	1.2 ± 0.5	1.3 ± 0.4	1.1 ± 0.4	1.1 ± 0.4	<0.01
EPI*, g/Kg/d^#^*	1.1 ± 0.4	1.2 ± 0.4	1.0 ± 0.3	0.9 ± 0.4	<0.01
Total fat*, g*	55 ± 26	59 ± 29	52 ± 20	51 ± 23	0.03
Total fat *(% of energy)*	31 ± 10	32 ± 10	30 ± 10	31 ± 10	0.50
Saturated fat*, g*	9 ± 7	9.7 ± 7.5	7.9 ± 4.8	7.7 ± 5.8	0.03
Saturated fat *(% of energy)*	5 ± 3	5 ± 3	4 ± 3	4 ± 3	0.04
Trans-fatty acids*, g*^***†***^	0.3 (0.1–0.5)	0.3 (0.2–0.5)	0.3 (0.1–0.6)	0.2 (0.1–0.4)	<0.01
Trans-fatty acids*, (% of energy)*^***†***^	0.1 (0.0–0.3)	0.2 (0.1–0.3)	0.2 (0.1–0.3)	0.1 (0.1–0.2)	0.05
MUFA*, g*	12 ± 10	13 ± 11	11 ± 8	11 ± 9	0.12
PUFA*, g*	10 ± 7	11 ± 8	10 ± 6	10 ± 7	0.10
Carbohydrates, *g*	224 ± 88	229 ± 83	226 ± 92	211 ± 95	0.18
Carbohydrates *(% of energy)*	56 ± 11	55 ± 10	56 ± 11	55 ± 12	1.00
Cholesterol, *mg*	383 ± 198	383 ± 190	377 ± 184	388 ± 228	0.93
Fiber*, g*	10 ± 5	11 ± 6	9 ± 5	9 ± 5	0.03
Micronutrients					
Sodium, mg	2,701 ± 1,095	2,774 ± 1,032	2,775 ± 1,134	2,474 ± 1,162	0.01
Sodium, mg^$^	2,979 ± 1,189	3,025 ± 1,166	3,147 ± 1,083	2,624 ± 1,356	0.26
Potassium, mg	1803 ± 714	1912 ± 728	1,658 ± 591	1732 ± 777	0.05
Phosphorus, mg	913 ± 334	982 ± 341	843 ± 281	844 ± 344	<0.01
Calcium, mg	437 ± 216	469 ± 226	402 ± 183	409 ± 221	0.04
Magnesium	252 ± 123	276 ± 147	232 ± 83	224 ± 94	<0.01
Iron, mg	19 ± 8	20 ± 9	18 ± 7	17 ± 8	0.05
Zinc, mg	9 ± 3	9 ± 3	9 ± 3	9 ± 3	0.07
Vitamin A, μg	405 ± 225	414 ± 213	396 ± 231	396 ± 245	0.55
Vitamin B1, mg	0.8 ± 0.3	0.8 ± 0.3	0.8 ± 0.4	0.8 ± 0.3	0.63
Vitamin C, mg	118 ± 81	123 ± 87	101 ± 60	119 ± 86	0.56

DEI, daily energy intake; DPI, daily protein intake; EPI, estimate protein intake; MUFA, monounsaturated fatty acids; PUFA, polyunsaturated fatty acids. ^#^DPI was calculated by 24-h urine urea nitrogen (UUN), with a sub-sample *n* = 232. ^$^Calculated from 24-h urine sodium excretion, with a sub-sample *n* = 130. ^†^Data were presented as Median (interquartile range).

The mean sodium intake calculated from dietary record and 24-h urine sodium excretion were 2,701 ± 1,095 mg and 2,979 ± 1,189 mg, respectively. Both values were higher than current recommendations. Men tended to consume more sodium than women (*p* = 0.02). Above findings confirmed that a large proportion of patients (71%) had their daily sodium intakes above the recommended level. A decreasing trend was observed in potassium and phosphorus intake across the CKD stages (*p* < 0.05).

### 3.3 Dietary quality

**Figure 2:**
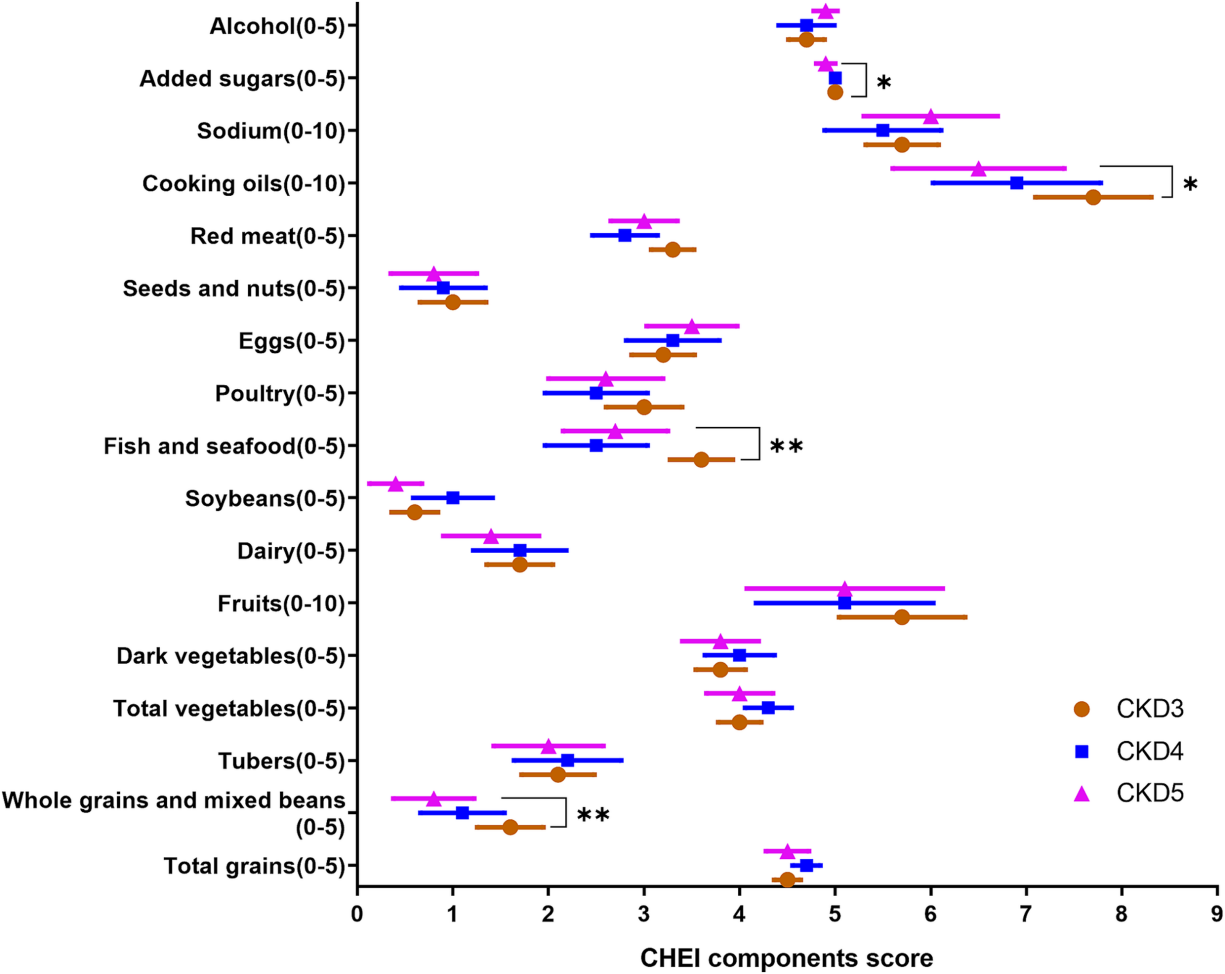
The components of CHEI score across stages of CKD severity; CHEI, Chinese Healthy Eating Index; *p < 0.05, **p < 0.01, *P* from Cuzick trend test.

Table 4 describes the dietary quality score according to CHEI. Figure 2 and Supplementary Figure 3 present the CKD stage-and sex-stratified CHEI food components score. Supplementary Figure 4 presents the proportion of each food group contributing to total protein and energy intake. The overall CHEI score was 59.5 ± 11.0 in all CKD patients, but patients with CKD stages 4 and 5 scored progressively worse (*P* trend <0.01). The component scores for whole grains, dairy, soybeans, tubers, and nuts were quantitatively low. Further, CKD-3 patients exhibited higher scores for whole grain, fish/seafood, and cooking oils, which represented a decreasing trend across the CKD stages (*p* value <0.05). Female patients exhibited higher scores for fruit intake and alcohol consumption (both *p* < 0.01). Total grains and tubers, red meat, and fish/seafood contributed 30%, 21%, and 12% of total protein intake, respectively, and 50%, 13%, and 10% of total energy intake, respectively.

**Table 4:** Evaluation of dietary quality by the CHEI and number of processed foods, overall and by stages of CKD severity.

	Total (*n* = 261)	CKD-3 (*n* = 130)	CKD-4 (*n* = 67)	CKD-5 (*n* = 64)	*P* trend
Total CHEI score	59.5 ± 11.0	61.3 ± 10.9	58.6 ± 11.1	56.9 ± 10.8	<0.01
Food processing, (%TEI)^†^					
PF and UPF	23.3 (12.4–33.0)	25.1 (14.9–35.0)	20.1 (13.6–30.3)	19.2 (8.0–29.8)	<0.01
PF	9.1 (1.9–17.4)	9.9 (3.3–18.4)	8.9 (0–17.4)	8.3 (0.5–15.7)	0.62
UPF	9.6 (0.6–18.6)	11.5 (3.9–22.2)	10.2 (0.3–16.3)	5.7 (0.2–12.4)	<0.01
Food processing, (%Weight)^†^					
PF and UPF	11.7 (6.0–17.7)	14.0 (6.9–19.9)	11.0 (5.6–15.4)	9.7 (5.0–16.3)	<0.01
PF	4.0 (0.8–7.7)	4.6 (1.4–8.6)	2.9 (0–6.3)	3.4 (0.4–7.5)	0.55
UPF	3.7 (0.8–7.4)	5.5 (1.4–9.0)	3.8 (0.4–6.5)	2.4 (0.3–5.7)	<0.01

CHEI, Chinese healthy eating index; IQR, interquartile range; TEI, total energy intake; PF, processed food; UPF, ultra-processed food. ^†^Data were presented as Median (IQR).

Table 4 shows the proportion of energy and weight attributed to PF and UPF. PF and UPF contributed 23.3% to total dietary energy and 11.7% to total dietary weight. CKD3 patients consumed more UPF, and patients with CKD4 and CKD5 consumed progressively less (*p* value <0.05).

## 4 Discussion

In this study, we evaluated the daily intake of patients with NDD-CKD stages 3–5 from our hospital and observed that a large proportion of patients had low adherence to Chinese renal diet recommendations, with excess protein and sodium as well as low energy and fiber intake. Some indicators of dietary quality were worse across more severe baseline categories of CKD. Specifically, there were lower intakes of whole grains, dairy, and soybeans, while the consumption of PF and UPF was relatively low. Female patients exhibited better diet quality than male patients, characterized by higher fruit consumption and lower intake of salt and alcohol.

Maintaining adequate energy intake is necessary to avoid PEW risks. With a mean DEI of 30 kcal/kg/d, about two-thirds of our patients failed to meet the minimal recommendations. The low energy intake aligns with findings from previous studies conducted in the Chinese population, where the average DEI ranged from 23 to 28 kcal/kg/d in NDD-CKD patients (39–41). The low DEI was partly explained in our study by the 13% of patients with signs of PEW at baseline. Chinese dietary habits may be another explanation for the low DEI (42), we showed that total grains and tubers supplied half of the total energy, and the consumption of large amounts of such lowly energy-dense foods may contribute to low energy intake.

The restriction of protein intake in advanced CKD is considered a key strategy to postpone dialysis, also by Chinese guidelines. Our study showed however that most patients consume more protein than recommended, with a slightly lower adherence rate than a previous Chinese study (41), which is consistent with current societal trends. Although we offer dietary counseling at our clinic, these results evidence non-compliance, which could be explained by insufficient or not clear enough advice, a lack of patient belief in the value of dietary restrictions, difficulties adhering to low protein intake regimes, or both. Furthermore, almost half of our patients were above 60 years old. At older ages, it may be more challenging to alter their dietary habits (43). Based on these, we are planning reinforcement strategies to improve adherence to DPI targets in our hospital.

Recent studies have awakened the interest in fiber intake and its potential benefits in reducing uremic toxicity production and inflammation, alleviating constipation, and being associated with a lower risk of adverse events (44, 45). Consistent with other reports from China (46, 47), most of our patients reported too little consumption of fiber in their diets, particularly in patients with more advanced CKD, possibly due to the long-rooted recommendation to restrict the consumption of fruits and vegetables in an attempt to prevent hyperkalemia. However, it seems that dietary potassium is not as strong a contributor to serum potassium levels as we initially assumed (48), and the KDOQI 2020 updates suggest that a more liberal consumption of low-potassium fruits and vegetables may be feasible and healthy in these patients (9). Further research should be done to confirm this point.

Consistent with the traditional profile of Chinese diets, which provide more than 3,000 mg of sodium per day (49, 50), most of our patients consumed more salt than recommended. Because it is difficult to quantify salt intake from food records, we also measured 24-h urinary sodium excretion, which provided consistent results. Although patients with more advanced CKD did consume less salt than patients in CKD stage 3, still too many exceeded the recommended thresholds. Recently, a large trial in rural China showed that substituting sodium chloride for potassium chloride in cooking salts reduced stroke, major adverse cardiovascular events, and all-cause death risk (51). Whether this strategy would be useful for patients with advanced CKD without increasing hyperkalemia risk is yet unknown and warrants evaluation.

Diet quality was progressively worse across more severe CKD stages, possibly as a consequence of the dietary restrictions needed. Specifically, we observed a relatively low intake of whole grains, dairy, and soybeans. By reducing animal sources of protein, carbohydrates become an important source of energy in the diet of our patients, with total grains contributing to 30% of protein intake. To meet the requirements of Chinese patients with traditional grain-based diets, low-protein rice, and whole grains can be used to substitute part or the entire amount of rice or cereal consumption. This may be particularly useful for patients with advanced/end-stage kidney disease, that is, increasing the intake of soybeans or their products and decreasing the intake of red meat.

We observed the consumption of PF and UPF was low compared to studies from Western populations (18, 52, 53). Although the current Chinese diet is nowadays more westernized, it is still a diet rich in fresh foods and with a strong basis on plants and cereals, particularly among older generations (54). Two general population studies from China show a similar proportion of UPF in the diet, contributing to about 10% of total energy (55, 56). Another reason for the relatively low consumption of UPF may be the nutrition campaigns currently ongoing and heavily publicized advocating for the consumption of fresh foods.

Strengths of this study include the use of a 3-day dietary record and the exclusion of under and over reporters. In addition, the 24-h urine sodium excretion and 24-h urine urea nitrogen (UUN) were collected to provide a more accurate estimation of dietary intake. The novelty is the evaluation of Chinese patients with CKD stages 3–5, a population with void of evidence in this aspect. As a clinical implication, our findings are a call for action on reinforced nutrition counseling strategies and patient support that correct the deficiencies hereby identified. This study also has limitations. First, it is a single-center study from an urban area in southern China and may not be extrapolated to other regions in China. Second, the use of a diet index developed for the general population to assess the quality of the diet for patients with CKD may have some limitations because it does not take into consideration the specific requirements of the disease. However, the overall diet quality evaluated by CHEI represents a dietary pattern composed of an adequate number of fruits, vegetables, and cereals and a lower number of red meats, sodium, and fat, which, collectively, also represents a healthy dietary pattern for CKD patients. Third, this study’s cross-sectional design limits our ability to infer causality in the associations observed.

NDD-CKD patients in our clinic have a dietary intake inconsistent with current dietary recommendations for this disease. Specifically, we observed excess protein and sodium intake, together with low energy and fiber. Although the consumption of UPF was relatively low compared to Western countries, the quality of the diet of our patients was progressively worse across more severe baseline CKD stages. Our results emphasize the need for a better and more flexible nutrition counseling strategy that not only focuses on micro/macronutrient intake but also on a diet of quality that aligns with traditional Chinese foods.

## Data Availability

The original contributions presented in the study are included in the article/Supplementary material, further inquiries can be directed to the corresponding author.
