# Supplementary figures and images for: Dietary quality and adherence to dietary recommendations in Chinese patients with chronic kidney disease
Source: Front Nutr. 2025 Feb 3;12:1547181. doi: 10.3389/fnut.2025.1547181 (PMC11831048; doi:10.3389/fnut.2025.1547181)

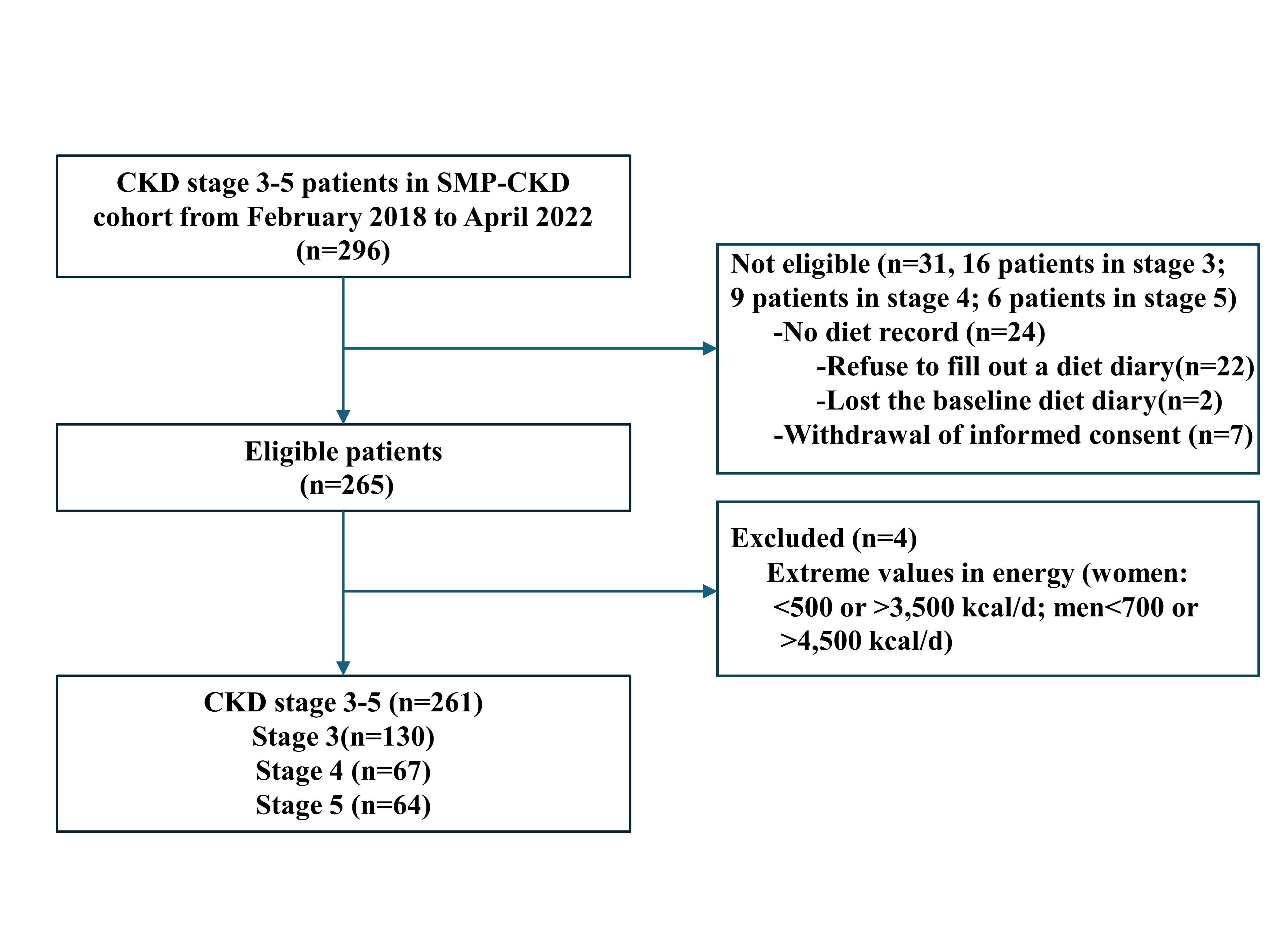

Supplement: Supplementary file 1 [file Image_1.TIF]

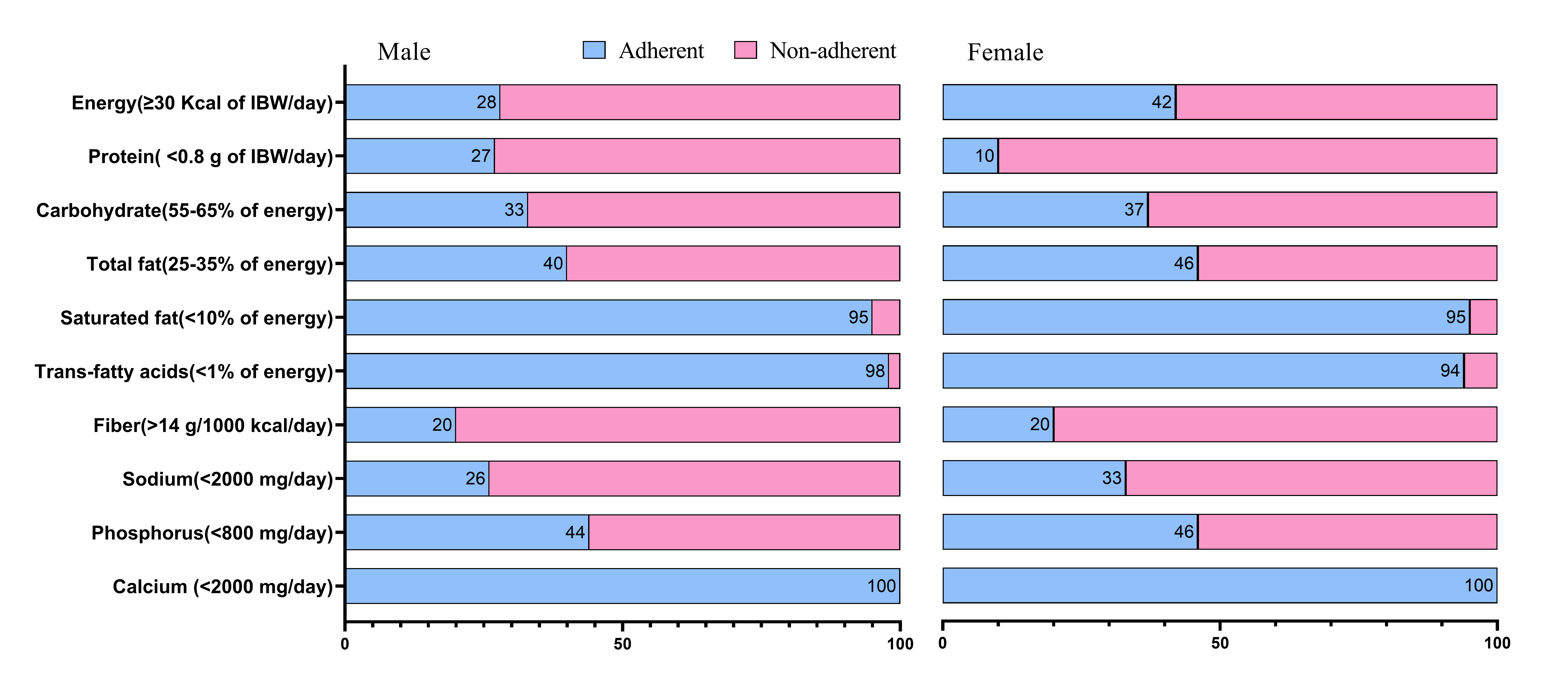

Supplement: Supplementary file 2 [file Image_2.TIF]

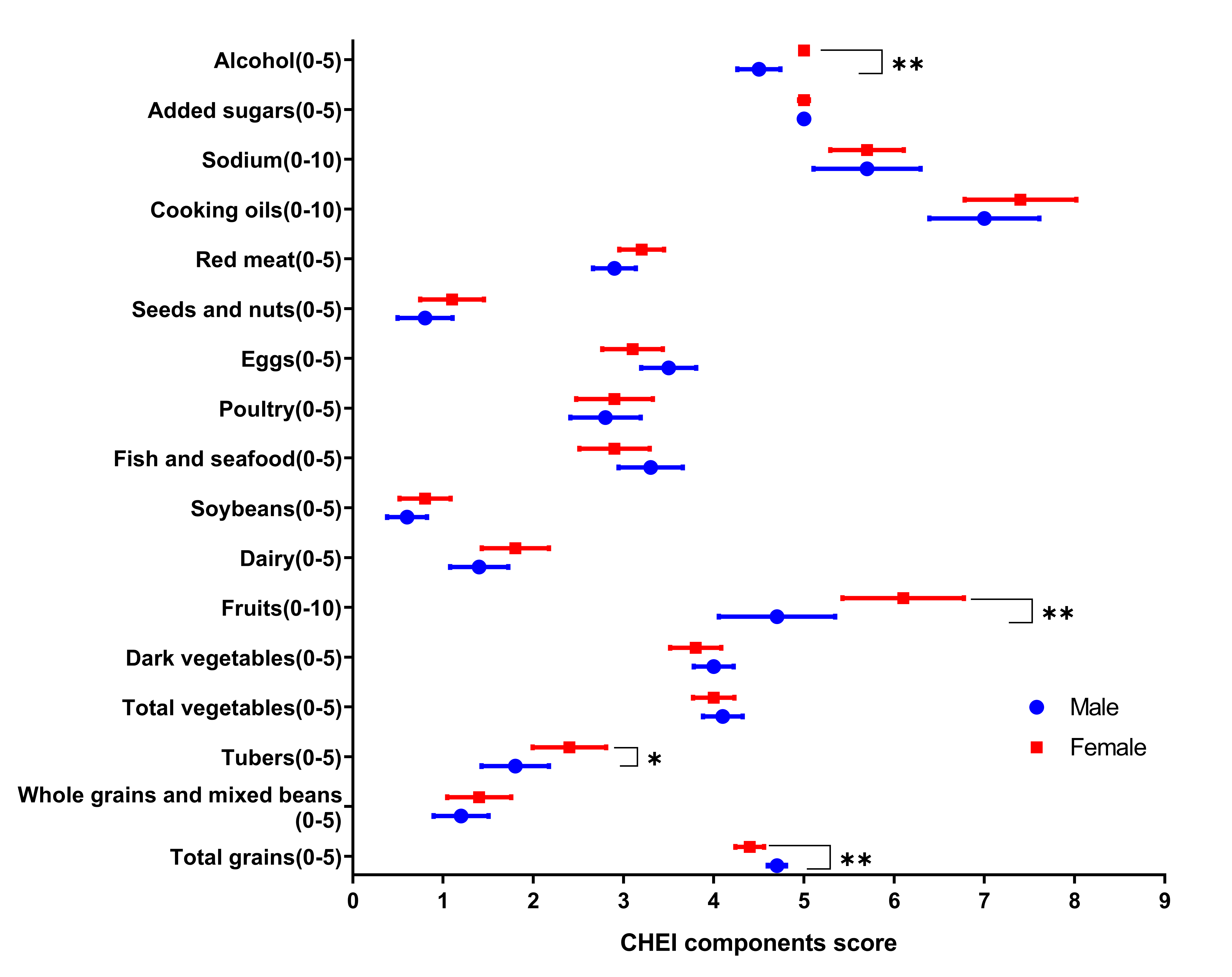

Supplement: Supplementary file 3 [file Image_3.TIF]

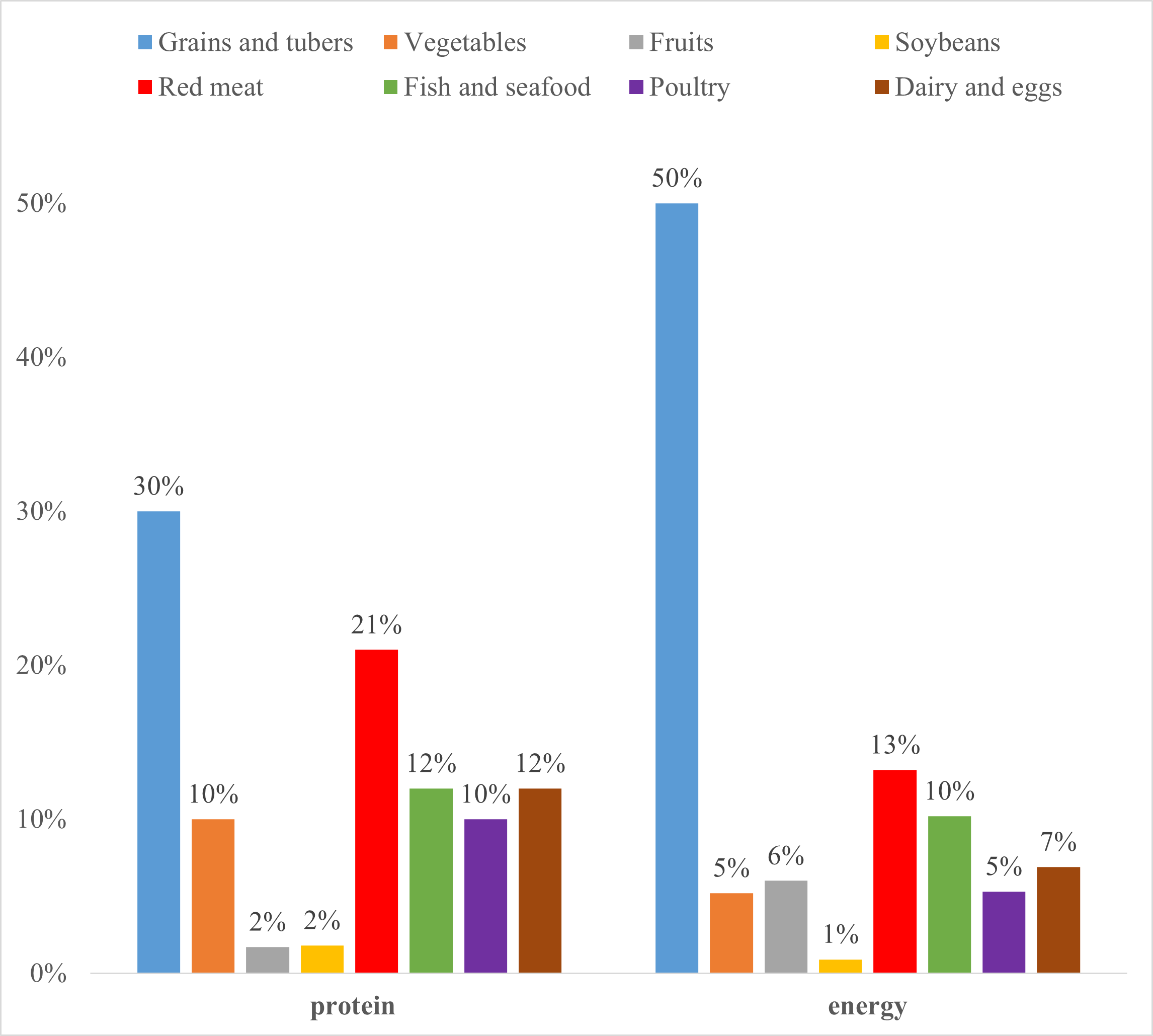

Supplement: Supplementary file 4 [file Image_4.TIFF]
