# Supplementary material for: Dietary quality and adherence to dietary recommendations in Chinese patients with chronic kidney disease
Source: Front Nutr. 2025 Feb 3;12:1547181. doi: 10.3389/fnut.2025.1547181 (PMC11831048; doi:10.3389/fnut.2025.1547181)
Supplement: Supplementary file 5 [file Table_1.DOCX]

Supplementary Material

# Supplementary Figures and Tables

## Supplementary Figures

**Supplementary Figure legends:**

Supplementary Figure 1 Flow chart of this study

Supplementary Figure 2 Proportion of patients adhering to the Chinese Dietary Guide for Chronic Kidney Disease stratified by sex. Data are expressed as percentage.

Supplementary Figure 3 The CHEI components score stratified by sex; CHEI: Chinese Healthy Eating Index; **P*<0.05, ***P*<0.01, P was calculated by Student-t test or Mann-Whitney U test.

Supplementary Figure 4 Contribution of food groups to the total protein and energy intake.

## Supplementary Tables

**Supplementary Table 1**: CHEI components, scoring criteria and standard portion size example

| Food components | Score range | scoring standard | Example food |
| --- | --- | --- | --- |
| **Adequacy** |  |  |  |
| Total grain | 0-5 | 0: no intake  5:≥2.5 SP/1000 kcal | rice(raw), Wheat flour, noodles(dry),  1SP=50g rice(raw)=125g rice(steamed) |
| Whole grains and mixed beans | 0-5 | 0: no intake  5:≥0.6 SP/1000 kcal | oatmeal, corn, millet  1SP=50g millet |
| Tubers | 0-5 | 0: no intake  5:≥0.3 SP/1000 kcal | potato, sweet potato, Taro  1SP=100g potato |
| Total vegetables | 0-5 | 0: no intake  5:≥1.9 SP/1000 kcal | pumpkin, sprouts, tomato  1SP=100g tomato |
| Dark vegetables | 0-5 | 0: no intake  5:≥0.9 SP/1000 kcal | cabbage, lettuce, spinach  1SP=100g spinach |
| Fruits | 0-10 | 0: no intake  10:≥1.1 SP/1000 kcal | apple, pear, banana  1SP=100g apple |
| Dairy | 0-5 | 0: no intake  5:≥0.5 SP/1000 kcal | milk, cheese, yogurt  1SP=200ml milk(liquid) |
| Soybeans | 0-5 | 0: no intake  5:≥0.4 SP/1000 kcal | Soybean, soybean curd, soybean milk  1SP=20g soybean=60g soybean curd |
| Fish and seafood | 0-5 | 0: no intake  5:≥0.6 SP/1000 kcal | grass carp, shrimp, hairtail  1SP=50g grass carp |
| Poultry | 0-5 | 0: no intake  5:≥0.3 SP/1000 kcal | chicken, duck, goose  1SP=50g chicken meat |
| Eggs | 0-5 | 0: no intake  5:≥0.5 SP/1000 kcal | egg, duck eggs  1SP=50g egg |
| Seeds and Nuts | 0-5 | 0: no intake  5:≥0.4 SP/1000 kcal | peanut, chestnut, Walnut  1SP=10g peanut |
| **Limitation** |  |  |  |
| Red meat | 0-5 | 0:≥3.5 SP/1000 kcal  5:≤0.4 SP/1000 kcal | lean pork, beef, sausage  1SP=50g lean pork=25g pork belly |
| Cooking oils | 0-10 | 0:≥32.6g/1000 kcal  10:≤15.6g /1000 kcal | peanut oil, sesame oil, olive oil |
| Sodium | 0-10 | 0:≥3608mg/1000 kcal  10:≤1000mg/1000 kcal | salt |
| Added sugar | 0-5 | 0:≥20% of energy  5:≤10% of energy | Cola, chocolate, dessert |
| Alcohols | 0-5 | 0:≥60g(male)/40g(female)  5: ≤25g(male)/15g(female) | beer, vodka, wine |

^*^ From 2016 Dietary Guidelines for Chinese (DGC-2016)

SP: standard portion

**Supplementary Table 2** Daily Intakes of Macronutrients and Micronutrients by sex

| Nutrients | Male (n=136) | Female (n=125) | *P* value |
| --- | --- | --- | --- |
| *Macronutrients* |  |  |  |
| Energy, *kcal* | 1701±518 | 1495±438 | <0.01 |
| DEI, kcal/kg/d | 28±8 | 32±9 | <0.01 |
| Total protein*, g* | 65±22 | 59±19 | 0.04 |
| DPI*,* *g/Kg/d* | 1.1±0.4 | 1.3±0.4 | <0.01 |
| EPI*, g/Kg/d^#^* | 1.0±0.4 | 1.1±0.3 | 0.04 |
| Total fat*, g* | 60±29 | 50±21 | <0.01 |
| Total fat *(% of energy)* | 32±10 | 31±10 | 0.46 |
| Saturated fat*, g* | 10±7 | 8±5 | 0.44 |
| Saturated fat *(% of energy)* | 5±3 | 4±3 | 0.26 |
| Trans-fatty acids*, g* ***^†^*** | 0.3(0.1-0.5) | 0.2(0.1-0.5) | <0.01 |
| Trans-fatty acids*, (% of energy)* ***^†^*** | 0.1(0.1-0.3) | 0.1(0.1-0.3) | 0.16 |
| MUFA*, g* | 13±11 | 10±8 | 0.01 |
| PUFA*, g* | 11±7 | 9±7 | 0.02 |
| Carbohydrates, *g* | 236±91 | 211±84 | 0.02 |
| Carbohydrates *(% of energy)* | 55±11 | 56±11 | 0.76 |
| Cholesterol, *mg* | 420±211 | 341±173 | <0.01 |
| Fiber*, g* | 10±5 | 10±6 | 0.69 |
| *Micronutrients* |  |  |  |
| Sodium, mg | 2847±1148 | 2541±1014 | 0.02 |
| Sodium, mg^$^ | 3268±1166 | 2715±1155 | <0.01 |
| Potassium, mg | 1809±713 | 1796±329 | 0.88 |
| Phosphorus, mg | 929±337 | 894±330 | 0.39 |
| Calcium, mg | 463±221 | 406±191 | 0.14 |
| Magnesium | 250±103 | 254±142 | 0.81 |
| Iron, mg | 20±8 | 18±8 | 0.06 |
| Zinc, mg | 10±3 | 9±3 | 0.01 |
| Vitamin A, μg | 428±255 | 380±185 | 0.08 |
| Vitamin B1, mg | 0.8±0.4 | 0.8±0.3 | 0.18 |
| Vitamin C, mg | 122±81 | 113±80 | 0.35 |

DEI: daily energy intake; DPI: daily protein intake; EPI: estimate protein intake; MUFA: monounsaturated fatty acids; PUFA, polyunsaturated fatty acids. *^#^ DPI was calculated by 24- hour urine urea* *nitrogen (UUN), with a sub-sample n=232. ^$^ Calculated from 24- hour urine sodium excretion, with a sub-sample n=130. ^†^* Data were presented as Median (interquartile range).

**Supplementary Table 3** Adherence to DEI and DPI recommendations by the presence or absence of PEW, n (%)

|  | Total | PEW  (n=35) | No PEW  (n=226) |
| --- | --- | --- | --- |
| Energy (≥30 Kcal of IBW/day) |  |  |  |
| *Overall* | 90(35) | 5(14) | 85(38) |
| *CKD3* | 54(42) | 4(27) | 50(43) |
| *CKD4* | 15(22) | 0(0) | 15(27) |
| *CKD5* | 21(33) | 1(10) | 20(37) |
| Protein (<0.8 g of IBW/day) |  |  |  |
| *Overall* | 50(19) | 7(20) | 43(19) |
| *CKD3* | 21(16) | 2(13) | 19(17) |
| *CKD4* | 17(25) | 1(10) | 16(28) |
| *CKD5* | 12(19) | 4(40) | 8(15) |
